# A comparative analysis of a disposable and a reusable pedicle screw instrument kit for lumbar arthrodesis: integrating HTA and MCDA

**DOI:** 10.1186/s13561-017-0153-7

**Published:** 2017-05-03

**Authors:** Claudia Ottardi, Alessio Damonti, Emanuele Porazzi, Emanuela Foglia, Lucrezia Ferrario, Tomaso Villa, Enrico Aimar, Marco Brayda-Bruno, Fabio Galbusera

**Affiliations:** 1 Laboratory of Biological Structure Mechanics, Department of Chemistry, Materials and Chemical Engineering “G. Natta”, Politecnico di Milano, Milan, Italy; 2 Centre for Research on Health Economics, Social and Health Care Management (CREMS), LIUC-Università, Cattaneo, Castellanza, Italy; 3 IRCCS Istituto Ortopedico Galeazzi, Milan, Italy

**Keywords:** Lumbar arthrodesis, Multi-criteria decision analysis, Health technology assessment, Reusable instrument, Disposable instrument, Italy

## Abstract

**Objective:**

Lumbar arthrodesis is a common surgical technique that consists of the fixation of one or more motion segments with pedicle screws and rods. However, spinal surgery using these techniques is expensive and has a significant impact on the budgets of hospitals and Healthcare Systems. While reusable and disposable instruments for laparoscopic interventions have been studied in literature, no specific information exists regarding instrument kits for lumbar arthrodesis. The aim of the present study was to perform a complete health technology assessment comparing a disposable instrument kit for lumbar arthrodesis (innovative device) with the standard reusable instrument.

**Methods:**

A prospective and observational study was implemented, by means of investigation of administrative records of patients undergoing a lumbar arthrodesis surgical procedure. The evaluation was conducted in 2013, over a 12- month time horizon, considering all the procedures carried out using the two technologies. A complete health technology assessment and a multi-criteria decision analysis approach were implemented in order to compare the two alternative technologies. Economic impact (with the implementation of an activity based costing approach), social, ethical, organisational, and technology-related aspects were taken into account.

**Results:**

Although the cost analysis produced similar results in the comparison of the two technologies (total cost equal to € 4,279.1 and € 4,242.6 for reusable instrument kit and the disposable one respectively), a significant difference between the two instrument kits was noted, in particular concerning the organisational impact and the patient safety.

**Conclusions:**

The replacement of a reusable instrument kit for lumbar arthrodesis, with a disposable one, could improve the management of this kind of devices in hospital settings.

## Background

Governments of developed countries, particularly in the western part of the World, have found it difficult to increase healthcare expenditure over the gross domestic product. Economic crises and the need to cut budgets have become more and more a priority on the policy makers agendas. This complex situation has been severe in Italy, where spending review austerity has been required, and strictly monitored by the Government [1].

As result, the provision, implementation and diffusion of expensive technology and organisational-based innovation [2] has been difficult and, often, limited. In this view, the technologies used in spinal surgery could be considered as part of the high-resource consumption innovations, considering both innovative techniques and consolidated interventions, such as spinal arthrodesis (the fixation of one or more spinal levels by means of pedicle screws and rods), assuming the Healthcare System or hospital point of view [3]. At institutional level in general, and in particular at hospital level, professionals and decision makers have introduced new devices, instruments and, in general, technologies only when they are able to demonstrate their value for money, in terms of technical advantages, effectiveness, equity and organisational aspects. Moving on from these premises, hospital clinicians and decision makers, as well as regional and national institutions could therefore adopt tools that are useful to assess the technologies-related evidence, for a better decision making process [4].

In this regard, several cost-effectiveness and cost-utility analyses have been conducted over the last 20 years, in Europe, with the aim of choosing the best treatment options for patients, without losing important financial resources [5, 6]. Health Technology Assessment (HTA) is a multi-dimensional and systematic approach useful to analyse different technologies, examining their economic, social, clinical, ethical and organisational implications [7]. The main objective of an HTA analysis is usually to create an evidence-based support for policy makers, optimising thus citizens’ health.

Reusable and disposable surgical instruments have been studied from a cost-effectiveness point of view, particularly with regard to laparoscopic surgical techniques: reusable instruments were found to be less expensive, ensuring the same performances in terms of safety and effectiveness [8, 9].

However, to the best of the authors’ knowledge, no studies have been focused on instrument kits in the specific setting of lumbar arthrodesis, with a complete multidimensional assessment approach. Therefore, the aim of the present study was to perform an HTA evaluation, focusing on two pedicle screws instrument kits used for short lumbar arthrodesis: in particular, an innovative disposable kit was compared with a traditional reusable one, with the development of a final multi-criteria decision analysis [10], thus being consistent with the EUnetHTA Core Model approach [11], EVIDEM suggestions [10] and Lombardy Region HTA practices [1].

## Methods

A Health Technology Assessment (HTA) approach was implemented, in order to achieve the primary objective of the study.

In particular, the HTA study was performed within a Spinal Surgery Unit of the “IRCCS Istituto Ortopedico Galeazzi” hospital of Milan. The standard reusable instrument kit used to perform a spinal stabilization by means of pedicle screws, was compared with the disposable device Steri Spine PS (Safe Orthopaedic SAS, Eragny-sur-Oise, France).

A prospective and observational study was implemented. The evaluation was conducted in 2013, over a 12 month time horizon, considering all the procedures carried out using both the technologies under investigation, by three experienced spinal surgeons.

Because of the multi-dimensional and multi-disciplinary nature of HTA, this approach considered several aspects of the medical technologies, as required by the EUnetHTA Core Model, 2016 [11]. In particular, the following dimensions were analysed (Table 1): *i)* general relevance; *ii)* safety; *iii)* efficacy and effectiveness; *iv)* economic and financial impact; *v)* equity; *vi)* legal aspects; *vii)* social and ethical impact; and *viii)* organisational impact. After the assessment of all the dimensions, a Multi-Criteria Decision Analysis (MCDA) approach [10] was implemented, thus defining the appraisal phase. First, as required by Lombardy Region HTA practise [1], the dimensions were prioritised, using a rating scale ranging from 1 (more important dimension) to 8 (least important dimension). Second, a three level rating score of 1 (less performant), 2 (equal performant) or 3 (more performant) was applied, by five expert evaluators, to each variable investigated, leading to a final concise result useful for the choice of the “preferable” technology.

**Table 1:** Methods used for the assessment of each dimension

Dimension	Method
General relevance	An in-depth literature review [14] was carried out, adapting the results obtained for the practices observed within the specific hospital involved in the study. The quality of scientific evidence and the agreement with the hospital policies were examined. This dimension required a description of the technology and the pathology under assessment and, in addition, the definition of the real and potential catchment area of reference for the procedures. In this view, evidence was retrieved using both Pubmed and Cochrane Databases, including the following keywords: “HTA assessment”, “surgical disposable devices”, “surgical instrument kit”. In particular, the literature review considered the following PICO: P (patients undergoing spinal surgical procedure), I (surgical disposable instrument kit), C (surgical reusable instrument kit), O (outcome related to the development of adverse events, economic data and clinical effectiveness).
Safety	The safety of patients (in terms of evaluation of adverse events, mortality or morbidity related to the technologies under assessment) and of healthcare professionals, as well as the environmental impact related to the use of the different technologies, was investigated using a 5-level Likert scale, ranging from a minimum of 1 (worst impact) to a maximum of 5 (best impact).
Efficacy and effectiveness	Since no comparative parameters of efficacy were found in the literature review, a qualitative indicator was collected from the real world evidence by means of a survey, directed at the experienced surgeons of the Clinical Institute performing these surgical procedures. This methodological approach was consistent with the literature of reference in the case of an absence of specific metrics and indicators [15]. The perceived effectiveness declared by the surgeons, using a 5-level scale ranging from a minimum of 1 (least performant), to a maximum of 5 (most performant), was retrieved, considering the ease of kit opening, the traceability, the comfort in use and the ease of identification of the product.
Economic and financial impact (assuming the hospital perspective)	• Activity Based Costing (ABC) analysis [16, 17]: an analysis that led to the identification, quantification, and consequent evaluation of resources required for a single clinical process, with the evaluation of all the direct costs (considering human resources, laboratory services, consumer products, and drugs, as well as sterilisation processes) related to each technology. In this view, the duration of each procedure and the level of the instrumentation, as well as the number of x-rays performed, were calculated in order to have a preliminary idea of the impact of the surgical procedure. Data regarding the pre-operative processes (order preparation time, supply time, storage area occupied, time, and cost of sterilisation) and intra-operative data (kit unpacking time, instrument preparation, and surgeons’ perceptions) were acquired. Here, it should be noted that all the information used for the proper economic assessment was directly retrieved from the hospital involved in the analysis. These aspects of expenditure were evaluated considering the 2014 Lombardy Region’s outpatients and hospital admission reimbursement tariff and drug costs, derived from the official NHS price list. • Cost-effectiveness analysis (CEA): an analysis correlated the above mentioned process costs (derived from the ABC) with the effectiveness data. • Budget Impact Analysis (BIA) [13]: a baseline scenario (historical) consisting only of patients undergoing the standard procedure (i.e. the reusable device) compared with an innovative scenario composed of the introduction and the implementation of the innovative technology.
Equity aspects	The health professionals involved in the study rated their perceptions, using a 7-item Likert scale, with regard to the accessibility, rapidity, usability, and invasiveness of the alternative technologies, as well as the access to care for persons of a legally protected status.
Legal impact	Both the indications of use for all the surgical procedures, categories of patients, and presence of authorization of use at national, European and International levels were evaluated using a 7-item Likert scale.
Ethical and social dimension	Usinging a 7-item Likert scale, the professionals involved evaluated the acceptability of the technologies under investigation, the impact of the technologies on the patient’s life style, the productivity losses impact, and the environmental impact and the impact of the procedure on the care giver’s life and perceptions.
Organisational impact	The organisational impact was evaluated using both a qualitative and a quantitative approach. From a quantitative perspective, all the additional investments required for the proper implementation of the technologies, as well as the impact of the innovative technology on the hospital processes, were investigated. From a qualitative point of view, the following items were assessed, considering the professionals’ perceptions: additional staff, training course, meetings and communication, learning curve, and equipment/furniture purchase or update.

A group, composed of three experienced surgeons using the investigated technologies and two individuals referring to the clinical management of the hospital of reference, was involved in the study, in order to obtain their qualitative perceptions.

## Results

### General relevance

The literature review revealed a lack of randomised controlled trials related to this topic, and also an absence of HTA reports and studies focusing on the comparison of the two instrument kits in the present study. In this view, only 2 papers (out of more than 25 evidence found, using the keywords “surgical disposable kit” and/or “HTA” and/or “spinal surgery”) met the research criteria, since the others were considered not focused on the efficacy or safety information, on the comparative analysis, out-of-scope for the evaluation or concerning other clinical specialties and not spinal surgery.

The debate concerning the use of reusable or disposable devices in the clinical practice is still open: in some cases, (e.g. laparoscopic interventions) it has been demonstrated that reusable instruments reduce the costs of the surgical treatments [8, 9] while disposable devices have been shown to have a positive influence on the hospital management, being easier to handle, to stock and not requiring a sterilization.

It emerged from the review that lumbar arthrodesis is a common surgical technique used to treat traumatic or degenerative pathologies and may be potentially performed on a large set of patients. The technologies involved in the HTA study were similar, in terms of the treatment indications and patients involved. Potential benefits of a disposable instrument kit are the reduction of the pre/post-operative costs due to the sterilization, the reduction of the risk of contamination, total traceability and new instruments for each surgery thus reducing the risk of damaged devices.

Focusing on the specific setting where the analysis was conducted, results showed that out of the 1,500 spinal surgeries performed every year, 139 were patients undergoing lumbar arthrodesis, thus revealing its importance (9%).

### Safety and effectiveness

The standard reusable technology is associated with a higher level of safety, even if the disposable instrument kits is advantageous for guaranteeing the safety of both healthcare professionals and patients. In quantifying the difference between the technologies, the standard one achieved a higher average value of perception when compared with the innovative one, even if the difference was not statistically significant (3.99 vs 3.70, *p*-value = 0.198).

With regard to effectiveness, the professionals involved in the analysis declared a final average score of 4.13 for the innovative kit compared with 3.56 for the reusable one (*p*-value = 0.035).

### Economic and financial impact

#### Activity based costing analysis

From the economic point of view, the implementation of an Activity Based Costing (ABC) approach was useful in order to highlight all the costs related to each technology. As reported in Table 2, the total cost estimated for the reusable instrument kit was € 4,279.1, while for the disposable kit it was € 4,242.6 (*p*-value =0.000).

**Table 2:** Economic evaluation of the technologies under assessment

		Reusable	Disposable
		(€)	(€)
Surgical procedure	Human Resources	1,092.5	1,092.5
	Laboratory services	115.9	115.9
	Consumer product	2,023.2	2,023.2
	Drugs	394.5	394.5
	Total cost	3,635.14^a^	3,626.1
	Total cost + VAT (17%)	4,253.1	4,242.6
Sterilisation	Total cost	21.4	-
	Total cost + VAT (20%)	25.7	
Total Process		4,279.1	4,242.6

Note: amortisation (^a^) of the devices must be accounted for during the year of purchase; thus, in case of reusable instruments, the amortisation is not considered in the HTA evaluation

#### Cost-Effectiveness analysis

Considering the effectiveness values previously mentioned, a Cost-Effectiveness Analysis (CEA) was performed: the two Cost-Effectiveness Values (CEVs) resulted in two indicators equal to 1,202.00 for the reusable kit, and 1,0278.26 for the disposable instruments: the lower the CEV related to a technology, the more preferable it is in comparison with the alternatives under investigation.

#### Budget Impact Analysis

In order to complete the economic and financial dimension, a Budget Impact Analysis (BIA) was implemented. The historical scenario, in which all the patients (N = 139) were treated using the standard reusable instrument kit, was compared with the innovative scenario, thus considering 119 procedures performed with the reusable instrument kit and 20 with the innovative disposable one. Over a 12-month time horizon, the historical scenario required economic resource absorption equal to € 594,797.68, whereas the innovative scenario considered a healthcare expenditure equal to € 594,067.08. The introduction of the innovative technology into clinical practice would result in an economic saving equal to −0.12% (−€ 730.60). A further scenario analysis was performed, comparing the historical scenario previously mentioned and the best-case scenario for the innovative technology (thus implementing it for all the 139 patients undergoing the surgical procedure): as result, the hospital of reference would decrease the healthcare expenditure devoted to the treatment of lumbar arthrodesis by −0.85% (−€ 5,077.67).

It should be noted that for the first year of implementation of the innovative technology, an additional amount of € 224.4 should be added as direct costs needed to ensure adequate staff training (additional organisational costs). Even if a budget holder considered the above mentioned organisational impact of the innovative technology introduction, the final result is positive and in favour of the innovative technology, thus representing a saving in costs, ranging from a minimum of −0.09%, in the worst case scenario, to a maximum of −0.82%, in the best case scenario.

### Other qualitative dimensions

**Fig. 1:**
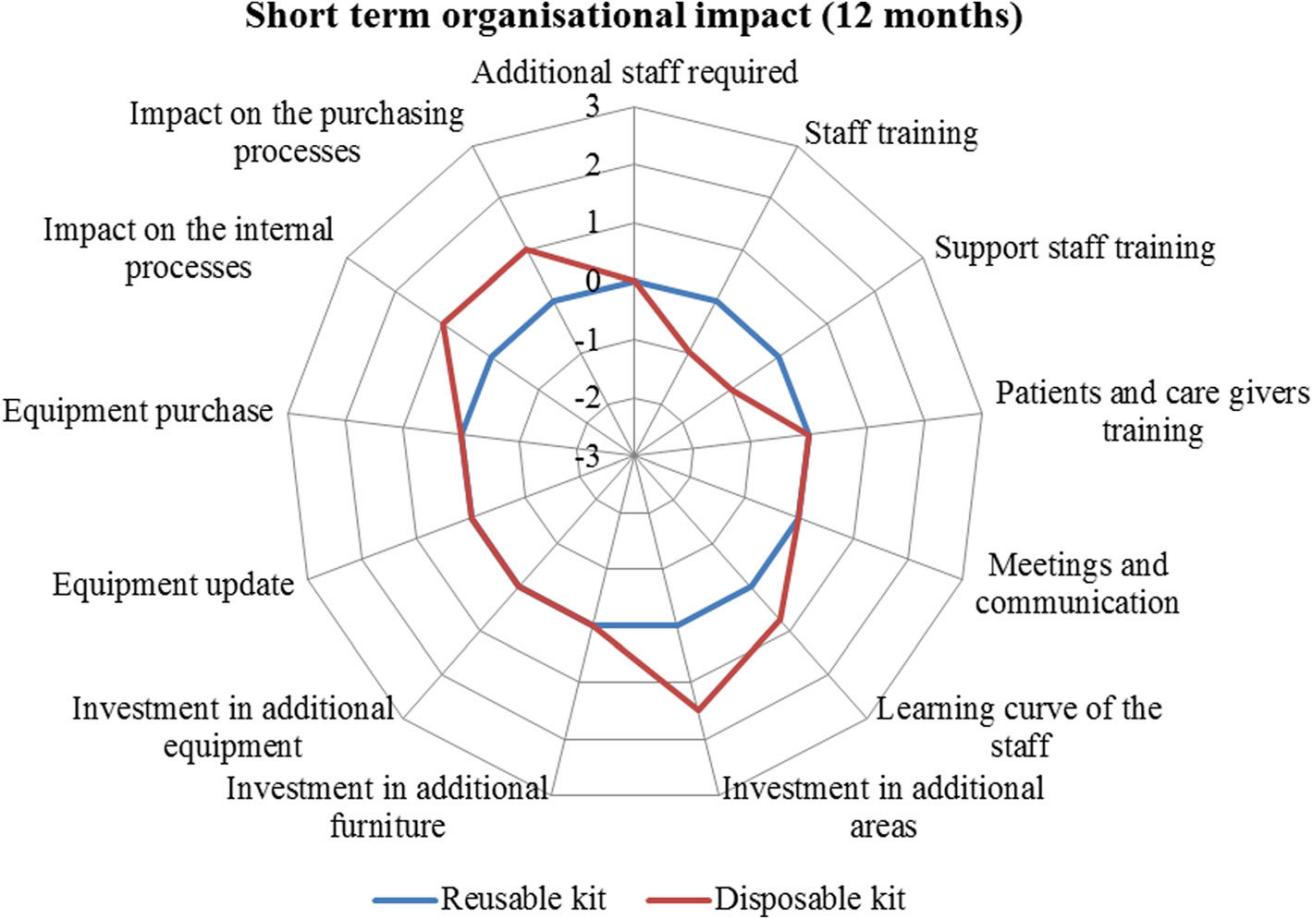
Organisational impact (12-month time horizon). Legend Section: The wider the area, the preferable the technology from an organisational point of view

With regard to the qualitative assessment of the organisational dimension, it emerged that, over a time-horizon of 12 months (Fig. 1), the introduction of the innovative technology requires the institutionalisation of specific training courses devoted to the healthcare professionals directly involved in the procedure on the one hand. On the other hand, it positively impacts, on both the internal and the purchasing processes, thus resulting in a general quantitative advantage if compared with the standard one (0.35 vs 0.00, *p*-value = 0.098).

**Fig. 2:**
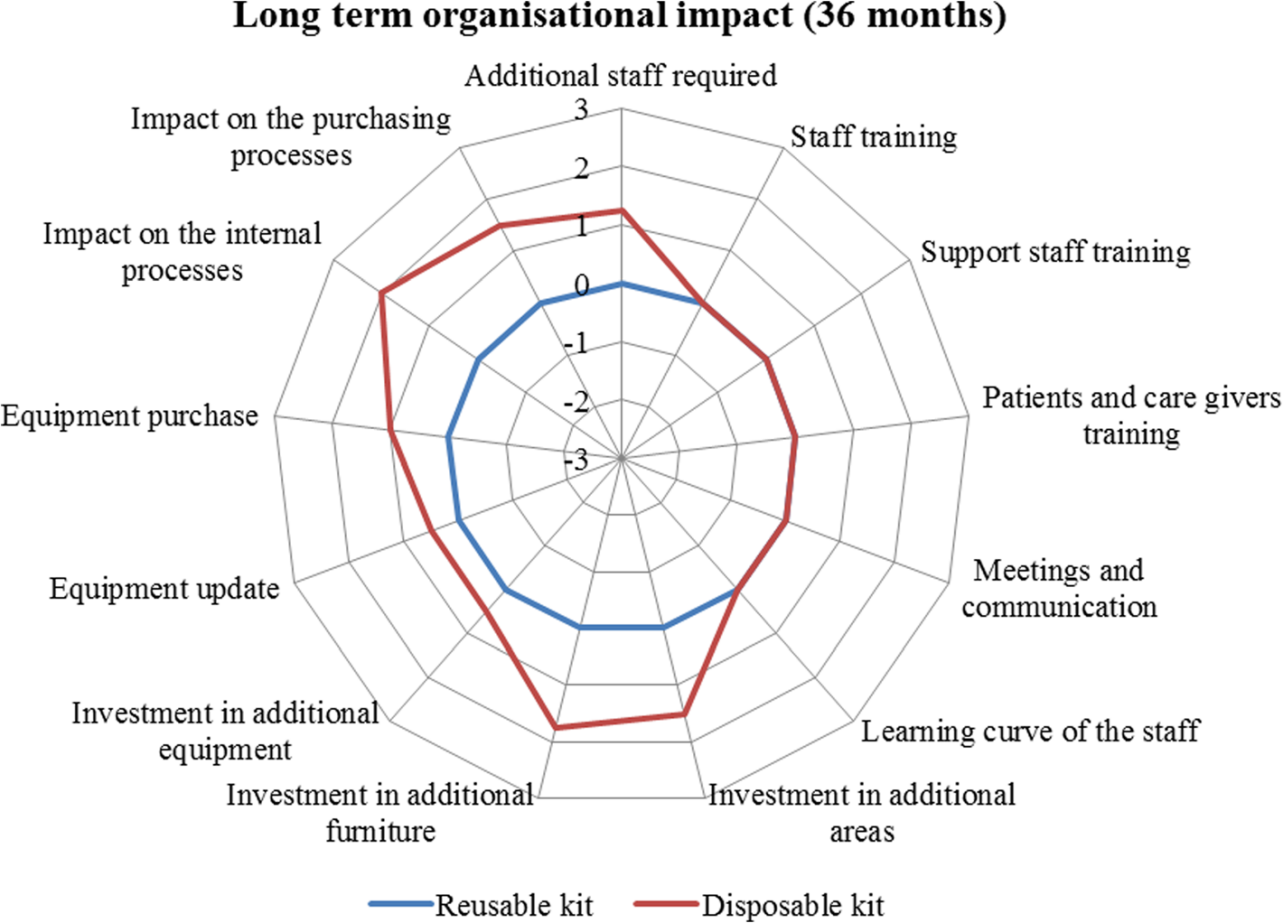
Organisational impact (36-month time horizon). Legend Section: The wider the area, the preferable the technology from an organisational point of view

Over a long-term time horizon (36 months, Fig. 2), the training could be completed, without requiring any additional resources. The innovative surgical kit positively influenced: *i)* equipment update; *ii)* equipment purchase; *iii)* internal processes, and *iv)* purchasing processes, in general. In quantitative terms, the innovative kit had a perceived overall organisational advantage with respect to the traditional one (0.77 vs 0.00, *p*-value = 0.003).

The analysis of the legal implications reported that the two technologies under assessment could be considered super-imposable in their measurement considering the indication of use for all the surgical procedures and for all the categories of patients, and the presence of authorisations of use.

**Fig. 3:**
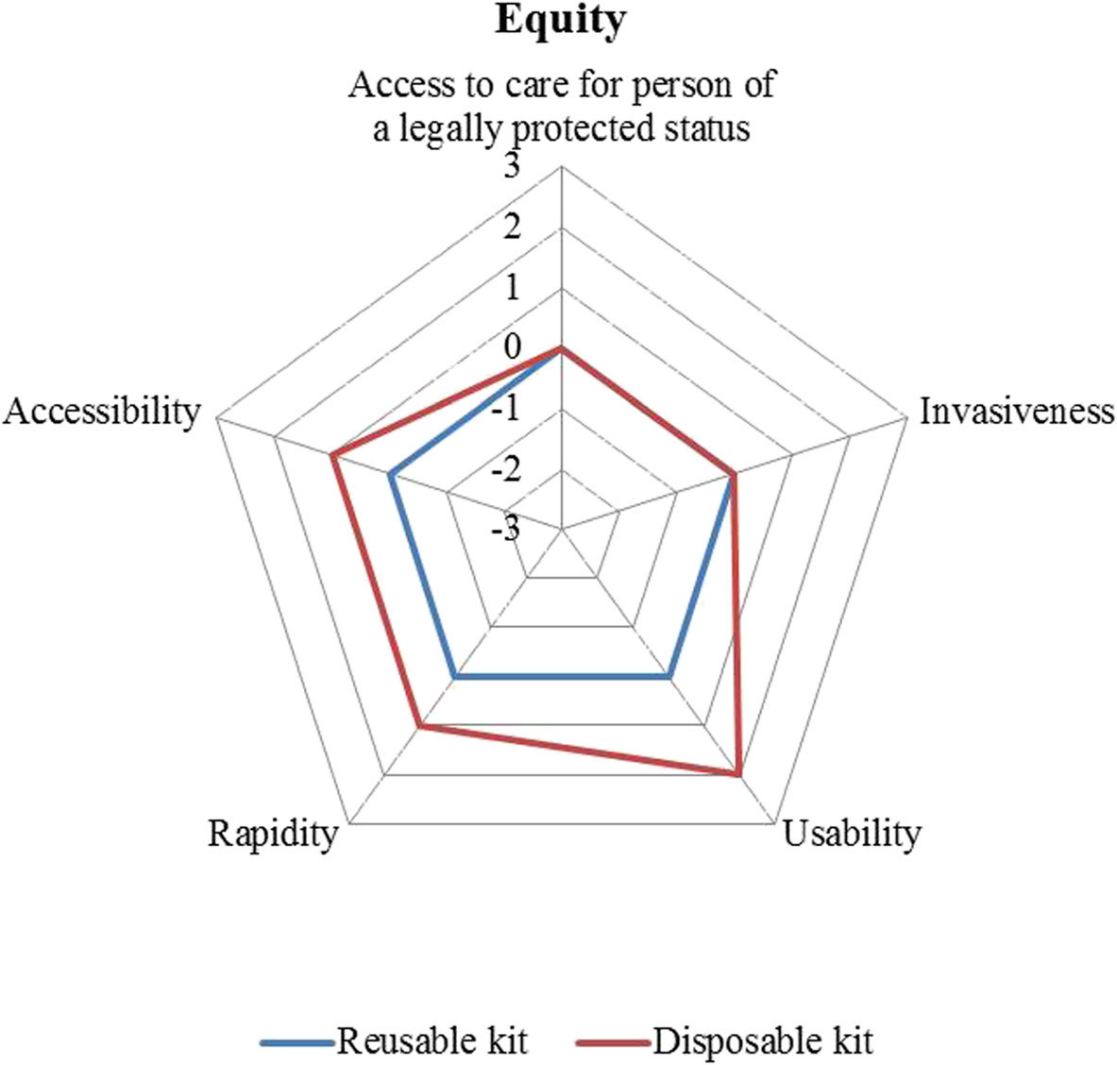
Equity impact. Legend Section: The wider the area, the preferable the technology from an equity point of view

Figure 3 reported the evaluators’ perception concerning the equity dimension. In particular, the innovative technology would lead to a significant decrease in waiting lists, thus improving the access to care in order to meet the citizens’ health needs. An incremental benefit related to the innovation resulted from the analysis of this dimension (0.80 vs 0.00, *p*-value = 0.011).

With regard to the ethical and social impact, clinicians declared that the patients could experience a positive impact from the use of the innovative technology due to the lower risk of developing future complications, thus improving the patient’s life style (0.00 vs 1, *p*-value = 0.007) However, the innovation generates a higher volume of waste, thus having a negative impact on the environment and leading to a worse perception on the social aspects (0.00 vs -0.67, *p*-value = 0.039).

Detailed results reporting the perceptions regarding safety, effectiveness, legal, equity, ethical, social and organisational dimensions are summarised in Table 3.

**Table 3:** Evaluators’ perceptions

Dimensions	Items	Reusable Kit		Disposable Kit	
Safety	Adverse events	4		4	
	Healtcare professionals’ safety	4		4.2	
	Patients’ safety	4		4.1	
	Environmental impact	3.95		2.5	
	Average value	3.99		3.70	
Effectiveness	Ease to opening the kit	4		4.2	
	Traceability	4.1		4.1	
	Surgeon comfort	4.2		3.8	
	Ease of identification of the product	1.95		4.4	
	Average value	3.56		4.13	
Legal impact	Indications of use for all the surgical procedures and for all the categories of patients	0		0	
	Presence of national, European and International authorizations of use	0		0	
	Average value	0		0	
Equity	Access to care for person of a legally protected status	0		0	
	Invasiveness	0		0	
	Usability	0		2	
	Rapidity	0		1	
	Accessibility	0		1	
	Average value	0		0.80	
Ethical impact	Acceptability of the technology	0		0	
	Impact of the technology on the patient life style	0		2	
	Average value	0		1	
Social impact	Impact on productivity losses	0		0	
	Environmental impact	0		−2	
	Impact of the procedure on the care givers lives and perceptions	0		0	
	Average value	0		−0.67	
		Reusable Kit	Disposable Kit	Reusable Kit	Disposable kit
		Short term	Short term	Long term	Long term
Organisational impact	Additional staff required	0	0	0	1.25
	Staff training	0	−1	0	0
	Support staff training	0	1.25	0	0
	Patients and care givers training	0	0	0	0
	Meetings and communication	0	0	0	0
	Learning curve of the staff	0	0.75	0	0
	Investment in additional areas	0	1.5	0	1.5
	Investment in additional furnitures	0	0	0	1.75
	Investment in additional equipment	0	0	0	0.5
	Equipment update	0	0	0	0.5
	Equipment purchase	0	0	0	1
	Impact on the internal processes	0	1	0	2
	Impact on the purchasing processes	0	1	0	1.5
	Average value	0	0.35	0	0.77

### Appraisal phase

With respect to the last phase of the analysis, both prioritisation and determination of a final score were obtained, useful for an evidence-based decision making appraisal.

In Table 4, the summary of all the results is presented. With regard to prioritisation, the healthcare professionals involved in the analysis (*N* = 5) declared that the most important dimension was the effectiveness, followed by economic impact and patient safety.

**Table 4:** Schematic representation of the priorities and scores (base, normalized and final)

Priority	Normalized priority	Dimensions	Specific dimensions	Base score		Score	Incidence	Normalized score		Final score	
				Reusable	Disposable	MAX	(%)	Reusable	Disposable	Reusable	Disposable
1	0.222	Effectiveness (practical research)		2	2	4	100	0.50	0.50		
		Total		4				0.50	0.50	0.11	0.11
2	0.194		Process analysis	2	2	4	36	0.18	0.18		
		Economic and financial impact	CEA	1	2	3	27	0.09	0.18		
			BIA	2	2	4	36	0.18	0.18		
		Total		11				0.45	0.55	0.09	0.11
3	0.167	Patient safety		1	2	3	100	0.33	0.67		
		Total		3				0.33	0.67	0.06	0.11
4	0.139		Quantitative	1	2	3	50	0.17	0.33		
		Organizational impact	Qualitative	1	2	3	50	0.17	0.33		
		Total		6				0.33	0.67	0.05	0.09
5	0.111		Scientific papers quality	1	1	2	10	0.05	0.05		
			Description of the technology	1	2	3	15	0.05	0.10		
			Description of the pathology	2	2	4	20	0.10	0.10		
		General relevance	Real and potential audience	2	2	4	20	0.10	0.10		
			Goals (agreement with company strategy)	1	2	3	15	0.05	0.10		
			Potential benefits	2	2	4	20	0.10	0.10		
		Total		20				0.45	0.55	0.05	0.06
6	0.083	Legal aspects		1	1	2	100	0.50	0.50		
		Total		2				0.50	0.50	0.04	0.04
7	0.056		Ethical impact	1	2	3	50	0.17	0.33		
		Social and ethical impact	Social impact	2	1	3	50	0.33	0.17		
		Total		6				0.50	0.50	0.03	0.03
8	0.028	Equity		2	2	4	100	0.50	0.50		
		Total		4				0.50	0.50	0.01	0.01
		FINAL SCORE								0.43	0.57

Three evaluators, different from the ones performing the prioritisation and the assessment, after a careful analysis of the results, assigned to each dimension and sub-dimension a three level mark (equal to 1, 2, or 3), thus determining the base score, defined as the average value attributed by the evaluators to each sub-dimension and, therefore, to each dimension (the sum of the previously mentioned scores).

After the evaluation of the average score and the total score, the percentage of incidence of each sub-dimension was calculated as the average mark assigned by the evaluators, divided by the total possible score for the specific dimension. The normalised score was then determined by multiplying the average base score for the two comparators with the percentage of incidence.

The final score was obtained by multiplying the normalised score calculated for each dimension with the normalized value of priority. In this view, the reusable instrument kit resulted in a score of 0.43, while the disposable kit resulted in a score of 0.57, underling an advantage for acquiring the innovative technology.

## Discussion

The Activity Based Costing approach showed that the costs of the two instrument kits are very similar, even though differences were found in the sterilisation phase (that must be carried out only in the reusable devices) and the amortization costs supported for the reusable instrument kit, only considered for the first year of use of the technology, in line with the Italian administrative law (that requires the amortization of all the deferred costs, in the first year). The results from CEA and BIA highlighted a slight advantage for the innovative technology, in terms of CEV and costs savings.

Results reported a residual better performance of the innovative technology in terms of patients’ safety: this is amply justified in literature, reporting that a disposable instrumentation kit could reduce the incidence of surgical site infections [12], attributing this to a reduction in exposure to air-borne bacteria in the operating room.

With regard to the organisational impact, the introduction of the disposable instrument kit would lead to significant advantages in the internal processes in the operative units and the overall purchasing phase. Moreover, the reduced size of the disposable instrument kit could guarantee easier management inside the hospital, both in terms of internal transport and storage processes: the manufacturing company could ensure supply upon request, faster than the standard sterilisation process.

In addition, its introduction could lead to a reduction of waiting lists, due to the quick supply of the devices (albeit this aspect could be dependent on the agreements signed by supplier companies and the hospital of reference) and their usability.

The present study was proposed for a specific clinical area (lumbar arthrodesis) in order to overcome the lack of literature evidence with regard to the investigated topic. Therefore, the results reported may not be considered replicable by an external reader, due to the restricted number of evaluators involved and the small sample of reference (composed of only 139 patients undergoing the surgical procedure). The study involved five healthcare professionals, and all the surgical patients representatives for this procedure were analysed, thus suggesting completeness of the results, and allowing their replicability within all spinal surgery units presenting at least the same number of patients.

Another limitation of the study is the time horizon taken into account for the budget impact evaluation that, for a rapid decision making process, considered only a 12-month time horizon, This, however, was useful in the negotiation process with the hospital management board, in accordance with the hospital budgeting processes and periods [13].

In order to overcome these limitations, an update of the economic and financial impact is therefore required, thus considering the healthcare expenditure over a period of three years.

## Conclusions

The present study may be considered as an attempt to quantify all the implications related to the implementation of a disposable instrument kit within the specific setting of lumbar arthrodesis, while not focusing attention only on argumentations related to the cost-effectiveness trade off. Despite the lack of evidence in literature, this multi-dimensional evaluation proved that the two investigated technologies, albeit apparently super-imposable, present some differences that needed an in depth analysis.

The HTA approach developed, supported by the MCDA methodology showed weaknesses and strengths, useful for both scholars and practitioners. On one hand, the findings could help scholars to crystalize the urgent need of effectiveness data among the alternative technologies under investigation. On the other, practitioners could understand the reason why they are called to use different technologies and to update the surgical practises, even if they are considered to be safe and consolidated.

In an era of spending reviews and paucity of resources, all strategies that are able to prevent a higher economic burden for hospitals and to re-engineer internal processes should be evaluated, and then, if affordable and feasible, implemented.

## Abbreviation

ABC: Activity Based Costing Analysis
BIA: Budget Impact Analysis
CEA: Cost-Effectiveness Analysis
CEV: Cost-Effectiveness Value
HTA: Health Technology Assessment
MCDA: Multi-Criteria Decision Analysis
